# The First Mitochondrial Genome of the Sepsid Fly *Nemopoda mamaevi* Ozerov, 1997 (Diptera: Sciomyzoidea: Sepsidae), with Mitochondrial Genome Phylogeny of Cyclorrhapha

**DOI:** 10.1371/journal.pone.0123594

**Published:** 2015-03-31

**Authors:** Xuankun Li, Shuangmei Ding, Stephen L. Cameron, Zehui Kang, Yuyu Wang, Ding Yang

**Affiliations:** 1 Department of Entomology, China Agricultural University, Beijing, China; 2 Earth, Environmental & Biological Sciences School, Science & Engineering Faculty, Queensland University of Technology, Brisbane, Australia; International Atomic Energy Agency, AUSTRIA

## Abstract

Sepsid flies (Diptera: Sepsidae) are important model insects for sexual selection research. In order to develop mitochondrial (mt) genome data for this significant group, we sequenced the first complete mt genome of the sepsid fly *Nemopoda mamaevi* Ozerov, 1997. The circular 15,878 bp mt genome is typical of Diptera, containing all 37 genes usually present in bilaterian animals. We discovered inaccurate annotations of fly mt genomes previously deposited on GenBank and thus re-annotated all published mt genomes of Cyclorrhapha. These re-annotations were based on comparative analysis of homologous genes, and provide a statistical analysis of start and stop codon positions. We further detected two 18 bp of conserved intergenic sequences from *tRNA^Glu^-tRNA^Phe^* and *ND1-tRNA^Ser(UCN)^* across Cyclorrhapha, which are the mtTERM binding site motifs. Additionally, we compared automated annotation software MITOS with hand annotation method. Phylogenetic trees based on the mt genome data from Cyclorrhapha were inferred by Maximum-likelihood and Bayesian methods, strongly supported a close relationship between Sepsidae and the Tephritoidea.

## Introduction

The mitochondrion (mt), descended from an alpha-proteobacterium, is one of the fundamental eukaryotic organelles [1–3] and retains a remnant, bacterial-like genome. The mt genome has been an extensively used marker in phylogenetic studies across broad taxonomic scales [4–8] and in a wide range of taxa since mt genome sequences are often more phylogenetically informative than shorter sequences from individual genes commonly used for shallow or species-level studies [9–12]. Since the first insect mt genome was published in 1985, there has been a rapid accumulation of sequenced insect genomes. Insects have been comprehensively sampled at higher taxonomic levels and mt genomes are available from every insect order [2]. Diptera is one of the most extensively sequenced orders amongst the Insecta, with 93 complete or near-complete Diptera mt genome sequences available on GenBank (as of July 2014), including 57 cyclorrhaphan species (46 complete genomes, 10 near-complete genomes without full control regions, and one partial genomes) representing 13 families (Table 1).

**Table 1:** Summary of mitogenome sequences from Brachycera.

Family	Species	Published information	GenBank Accession No.	Length (bp)
Tabanidae	Trichophthalma punctata#*	[13]	NC_008755	16396
Nemestrinidae	Cydistomyia duplonotata#*	[13]	NC_008756	16247
Phoridae	*Megaselia scalaris*	-	NC_023794	15599
Syrphidae	*Simosyrphus grandicornis*	[13]	NC_008754	16141
Fergusoninidae	*Fergusonina taylori*	[14]	NC_016865	16000
Agromyzidae	*Liriomyza bryoniae*	[15]	NC_016713	16183
	*Liriomyza huidobrensis*	[15]	NC_016716	16236
	*Liriomyza sativae*	[16]	NC_015926	15551
	*Liriomyza trifolii*	[17]	NC_014283	16141
Tephritidae	*Bactrocera carambolae*	[18]	NC_009772	15915
	*Bactrocera correcta*	-	NC_018787	15936
	*Bactrocera cucurbitae*	-	NC_016056	15825
	*Bactrocera dorsalis*	[19]	NC_008748	15915
	*Bactrocera minax*	[20]	NC_014402	16043
	*Bactrocera oleae*	[21]	NC_005333	15815
	*Bactrocera papayae*	[18]	NC_009770	15915
	*Bactrocera philippinensis*	[18]	NC_009771	15915
	*Bactrocera tryoni*	[22]	NC_014611	15925
	*Ceratitis capitata*	[23]	NC_000857	15980
	*Procecidochares utilis*	-	NC_020463	15922
Drosophilidae	*Drosophila ananassae*	[24]	BK006336 (Without CR)	-
	*Drosophila erecta*	[24]	BK006335 (Without CR)	-
	*Drosophila grimshawi*	[24]	BK006341 (Without CR)	-
	*Drosophila littoralis*	[25]	NC_011596	16017
	*Drosophila melanogaster*	[26]	NC_001709	19517
	*Drosophila mojavensis*	[24]	BK006339 (Without CR)	-
	*Drosophila persimilis*	[24]	BK006337 (Without CR)	-
	*Drosophila pseudoobscura*	[27]	NC_018348 (Without CR)	-
	*Drosophila santomea*	[28]	NC_023825	16022
	*Drosophila sechellia*	[29]	NC_005780 (Without CR)	-
	*Drosophila simulans*	[29]	NC_005781 (Without CR)	-
	*Drosophila virilis*	[24]	BK006340 (Without CR)	-
	*Drosophila willistoni*	[24]	BK006338 (Without CR)	-
	*Drosophila yakuba*	[30]	NC_001322	16019
Sepsidae	*Nemopoda mamaevi*	Present study	KM605250	15878
	*Haematobia irritans*	-	NC_007102	16078
Muscidae	Musca domestica*	[31]	NC_024855	16108
	*Stomoxys calcitrans*	[32]	DQ533708	15790
Scathophagidae	Scathophaga stercoraria*	[31]	NC_024856	16223
Calliphoridae	*Calliphora vicina*	[5]	NC_019639	16112
	*Chrysomya albiceps*	[5]	NC_019631	15491
	*Chrysomya bezziana*	[5]	NC_019632	15236
	*Chrysomya megacephala*	[5]	NC_019633	15273
	*Chrysomya putoria*	[33]	NC_002697	15837
	*Chrysomya rufifacies*	[5]	NC_019634	15412
	*Chrysomya saffranea*	[5]	NC_019635	15839
	*Protophormia terraenovae*	[5]	NC_019636	15170
	*Cochliomyia hominivorax*	[34]	NC_002660	16022
	*Lucilia cuprina*	[5]	NC_019573	15952
	*Lucilia porphyrina*	[5]	NC_019637	15877
	*Lucilia sericata*	[5]	NC_009733	15945
	*Hemipyrellia ligurriens*	[5]	NC_019638	15938
Polleniidae	*Pollenia rudis*	[5]	JX913761 (Partial Genome)	-
Oestridae	*Dermatobia hominis*	-	NC_006378	16460
	*Hypoderma lineatum*	[35]	NC_013932	16354
Sarcophagidae	*Sarcophaga impatiens*	[14]	NC_017605	15169
	*Sarcophaga peregrina*	-	NC_023532	14922
Tachinidae	*Elodia flavipalpis*	[6]	NC_018118	14932
	*Exorista sorbillans*	[36]	NC_014704	14960
	*Rutilia goerlingiana*	[5]	NC_019640	15331

Note: “-” not available (unknown or incomplete data);

“*” species used in phylogenetic analysis;

“^#^” outgroup.

Sepsidae is a global distributed fly family with more than 320 described species [37]. Sepsid flies are important insect models for sexual selection research for three main reasons: 1. pronounced sexual dimorphisms (strongly modified male forelegs and movable abdominal appendages) [38–40]; 2. complex courtship behaviors (male display, female choice, and sexual conflict) [38, 41–43]; and 3. easily bred under lab conditions (they utilize rotting plant material or animal feces as breeding substrates) [44]. Recently, the transcriptome of a sepsid species *Themira biloba* has been assembled and analyzed [45] expanding the range of genetic resources for this family, however, no mt genomes are available from this family.

The procedures and software used for insect mt genomes annotation have recently been reviewed by Cameron (2014b) [3] noting that accurate annotations of mt genomes are necessary for all downstream analysis. Since the online implementation of the tRNA prediction software tRNAScan-SE [46] plus alignment with homologous genes is relatively efficient, there are few problems in identifying gene boundaries for tRNAs. However, despite protein-coding genes (PCGs) being used in virtually every phylogenetic and evolutionary biology study of mt genomes, gene boundaries of some PCGs are often difficult to identify. For example, the start codons of *CO1* are wildly inconsistent and there are some inaccurate annotations in the GenBank (e.g. 132 incorrect annotations across 36 species of lepidopteran mt genomes [3]). Studies of expression profiles of mt genes should be the most effective way to identify gene boundaries [12, 47–48], however there are few RNAseq datasets for insect species whose mt genomes have also sequenced. In the absence of RNAseq data, comparative alignments of homologous mt genes from all the mt genomes available for a particular taxonomic group is also reliable [3].

Here, we sequenced the complete mt genome of the sepsid fly *Nemopoda mamaevi* Ozerov, 1996. We annotated with this genome using procedures and quality control methods proposed by Cameron (2014b) [3] and compared these annotation results with the automated annotation software MITOS [49]. We also re-annotated the mt genomes of all Cyclorrhapha species deposited on GenBank, based on comparative analysis of homologous genes, and undertook a statistical analysis of start and stop codons positions in their PCGs. We aligned and analysed two intergenic sequences across Cyclorrapha, which were highly conserved 18-bp motifs for the binding site of mtTERM. The mt genome contributes to reconstruction of the taxonomic positions and evolutionary relationships of the Sepsidae, and will help selecting optimized primer for atypical regions in further molecular research of related taxa. Phylogenetic trees based on the mt genome data from Cyclorrhapha were inferred by both Maximum-likelihood and Bayesian methods, which strongly supported a close relationship between Sepsidae and the Tephritoidea.

## Material and Methods

### Ethics statement

No specific permits were required for the insects collected for this study. The specimen was collected by using light trap. The field studies did not involve endangered or protected species. The species herein studied are not included in the “List of Protected Animals in China”.

### Sampling and DNA extraction

The specimen used for DNA extraction was collected by Yuting Dai from Xiaolongmen (N39°57′55.21″ E115°27′59.58″), Mentougou, Beijing, China, in June 2013. After collection, it was initially preserved in 95% ethanol in the field, and then transferred to -20°C for the long-term storage upon the arrival at China Agricultural University. The specimen was examined and identified by the first author Xuankun Li with ZEISS Stemi 2000–c microscope. It is distinct from other *Nemopoda* by the absence of setae near the anterior margin of the katepisternum, presence of a large blackish apical spot on the male wing, and absence of setae on male hind femur [50]. *Nemopoda mamaevi* Ozerov is newly recorded from China. Whole genomic DNA was extracted from the thoracic muscle tissues using TIANamp Genomic DNA Kit (TIANGEN). The quality of PCR products was assessed through electrophoresis in a 1% agarose gel and stained with Gold View (ACME).

### PCR amplification and sequencing

The *Nemopoda mamaevi* mt genome was amplified in 20 overlapping PCR fragments using NEB Long Taq DNA polymerase (New England BioLabs, Ipswich, MA). The PCR primers used follow Zhao et al (2013) [6]. Several species-specific primers were designed from initial sequencing with the amplification primers and used for internal PCRs (S1 Table).

PCR cycling consisted of an initial denaturation step at 95°C for 30s, followed by 40 cycles of denaturation at 95°C for 10s, annealing at 42–55°C (depending on the primer pair used) for 50s, elongation at 65°C for 1 kb/min (depending on the size of target amplicon) (S1 Table), and a final elongation step at 65°C for 10 min. PCR products were evaluated by agarose gel electrophoreses.

All amplicons were sequenced in both directions using the BigDye Terminator Sequencing Kit (Applied Bio Systems) and the ABI 3730XL Genetic Analyzer (PE Applied Biosystems, San Francisco, CA, USA) using amplification primers and internal primers developed by primer walking.

### Bioinformatic analysis

Sequences were proof-read and aligned into contigs in BioEdit version 7.0.5.3 [51]. After fully sequencing the mt genome, it was annotated using both automated annotation methods and by hand. For the automated annotation, we used MITOS [49]. Hand annotation method followed the procedures proposed by Cameron (2014b) [3]. Quality control of the hand alignments was performed by comparing with homologous sequences from previously sequenced Cyclorrhapha mt genomes to identify several tRNAs apparently absent from the *N*. *mamaevi* mt genome, and to verify PCGs and rRNAs annotations.

Nucleotide substitution rates, base composition and codon usage were analyzed with MEGA 5.0 [52]. Nucleotide compositional skew was measured using the following formula: AT-skew = (A-T)/(A+T) [53].

### Phylogenetic analysis

A total of 20 species of brachyceran insects were used in phylogenetic analysis, including 18 cyclorrhaphans and two outgroup species from Tabanidae and Nemestrinidae. Details of the species used in this study were listed in Table 1.

Sequences of 13 PCGs, two rRNAs and 22 tRNAs were used in phylogenetic analysis. Each PCG was aligned individually based on Hand annotation method followed the procedures proposed by Cameron (2014b) [3]. The sequences of tRNAs and rRNAs were aligned respectively using MEGA 5.0 [52], ambiguous positions in the alignment of RNAs were filtered by hand. Individual genes were concatenated using SequenceMatrix v1.7.8 [54]. We assembled four datasets for phylogenetic analysis: 1) nucleotides of 13 PCGs (PCG123) with 11,058 residues, 2) nucleotides of 13 PCGs, two rRNAs and 22 tRNAs (PCG123RNA) with 14,226 residues, 3) nucleotides of 13 PCGs exclude the third codon sites (PCG12) with 7,372 residues and 4) nucleotides of 13 PCGs exclude the third codon sites, two rRNAs and 22 tRNAs (PCG12RNA) with 10,540 residues. PartitionFinder v1.1.1 [55] was used to select the optimal partition strategy and substitution models for each partition. We created an input configuration file with 63/29 (with 3^rd^ codon positions/ without) pre-defined partitions of the dataset, and used the ‘‘greedy” algorithm with branch lengths estimated as ‘‘unlinked” and Bayesian information criterion (BIC) to search for the best-fit scheme (S2 Table).

We performed maximum likelihood (ML) and Bayesian inference (BI) using the best-fit partitioning schemes recommended by PartitionFinder (S2 Table). For ML analysis, we used RAxML 8.0.0 [56] with 1,000 bootstrap replicates and using the rapid bootstrap feature (random seed value 12345) [57]. The Bayesian analysis was conducted with MrBayes 3.2.2 [58]. Two simultaneous runs of 2 million generations were conducted for the dataset, each with one cold and three heated chains. Samples were drawn every 1,000 Markov chain Monte Carlo (MCMC) steps, with the first 25% discarded as burn-in. When the average standard deviation of split frequencies was below 0.01, we considered the stationarity was reached and stopped run.

## Results and Discussion

### Genome Organization and Structure

**Fig 1:**
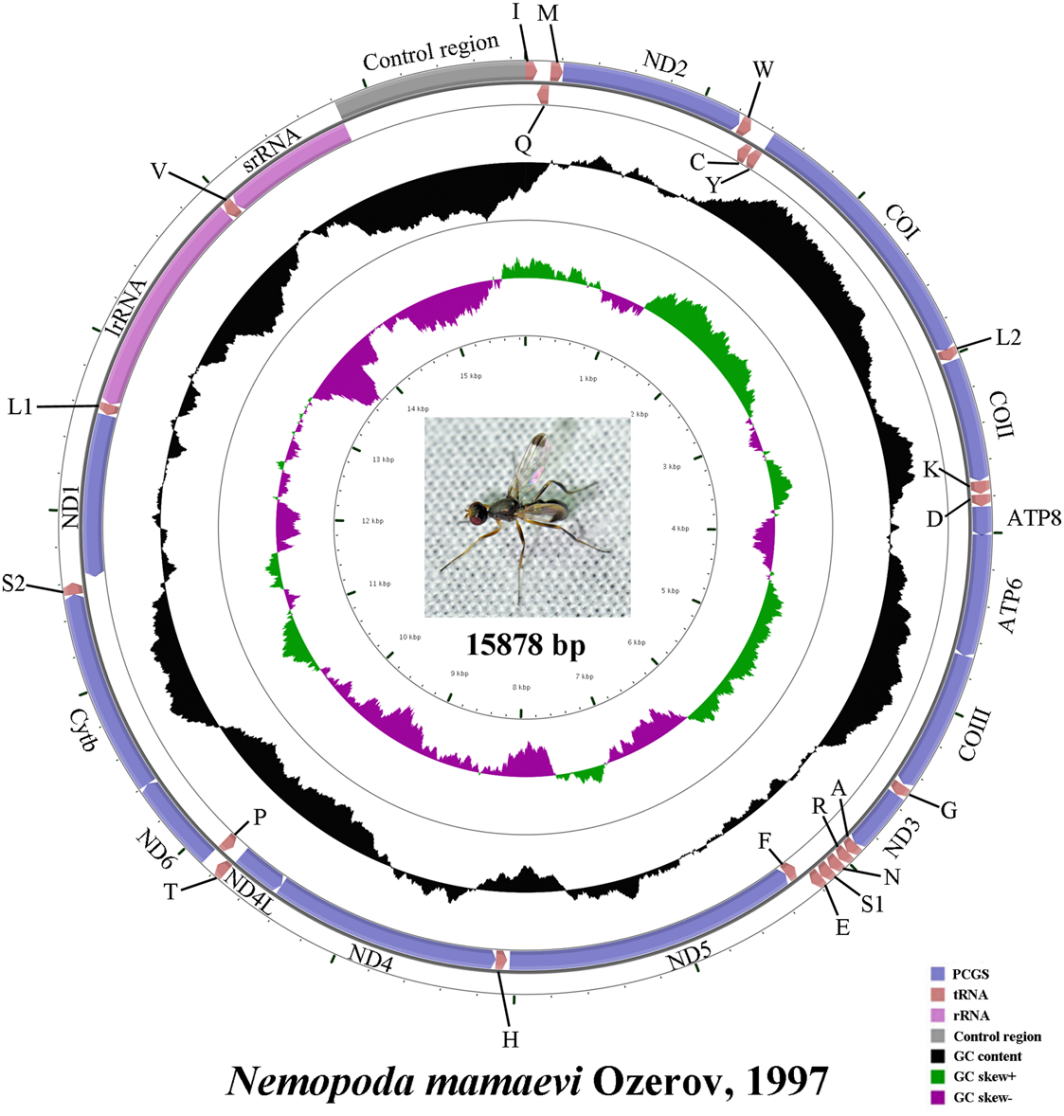
Mitochondrial map of *Nemopoda mamaevi* Ozerov. Circular maps were drawn with CGView [59]. Arrows indicated the orientation of gene transcription. The tRNAs are denoted by the color blocks and are labelled according to the IUPACIUB single-letter amino acid codes (L1: CUN; L2: UUR; S1: AGN; S2: UCN). The GC content was plotted using a black sliding window, as the deviation from the average GC content of the entire sequence. GC-skew was plotted as the deviation from the average GC-skew of the entire sequence. The inner cycle indicated the location of genes in the mt genome.

The complete mt genome of *N*. *mamaevi* is a typical circular, double-stranded molecule (GenBank accession number: KM605250; Fig 1) 15,878 bp in length. Mt genome length is medium-sized when compared to the mt genomes of other Diptera, that range from 14,922 bp (*Sarcophaga peregrine*, Sarcophagidae, NC_023794) to 19,517 bp (*Drosophila melanogaster*, Drosophilidae [26]). The mt genome of *N*. *mamaevi* contains the 37 genes, including 13 PCGs, 22 tRNA genes, two rRNA genes and a large control region, that are usually present in bilaterian animals [3]. The gene order is the same as the inferred ancestral insect mt genome pattern, which is conserved amongst all cyclorrhaphan flies sequenced to date. Twenty three genes are encoded on the majority strand (J-strand), while the minority strand (N-strand) encodes the remaining 14 genes.

There are 9 gene boundaries where sequence overlapped between neighboring genes, ranging from 1 to 8 bp in length, and 29 bp in total. The length of intergenic sequences (excluding the control region) was 1–18 bp found at 14 gene boundaries, and totalling 82 bp (Table 2). The longest overlapping sequence belonged to both *tRNA* ^*Trp*^ and *tRNA* ^*Cys*^. The longest intergenic sequence located between *tRNA* ^*Glu*^ and *tRNA* ^*Phe*^.

**Table 2:** Organization of the mitogenome of *Nemopoda mamaevi* Ozerov.

Gene	Direction	Location	Size(bp)	IGN*	Anticodon	Codon		AT%
						Start	Stop	
tRNA^Ile^	F	1–66	66		GAT			74.3
tRNA^Gln^	R	67–135	69	0	TTG			81.2
tRNA^Met^	F	140–208	69	4	CAT			68.1
*ND2*	F	209–1231	1023	0		ATT	TAA	74.8
tRNA^Trp^	F	1237–1303	67	5	TCA			73.1
tRNA^Cys^	R	1296–1359	64	-8	GCA			70.4
tRNA^Tyr^	R	1360–1426	67	0	GTA			74.6
*CO1*	F	1425–2963	1539	-2		TCG	TAA	65.8
tRNA^Leu(UUR)^	F	2959–3024	66	-5	TAA			74.2
*CO2*	F	3030–3713	684	5		ATG	TAA	70.6
tRNA^Lys^	F	3715–3785	71	1	CTT			64.8
tRNA^Asp^	F	3789–3855	67	3	GTC			86.6
*ATP8*	F	3856–4017	162	0		ATC	TAA	74.1
*ATP6*	F	4011–4688	678	-7		ATG	TAA	71.4
*CO3*	F	4688–5476	789	-1		ATG	TAA	65.7
tRNA^Gly^	F	5483–5547	65	6	TCC			78.5
*ND3*	F	5548–5901	354	0		ATC	TAG	75.7
tRNA^Ala^	F	5900–5964	65	-2	TGC			72.3
tRNA^Arg^	F	5964–6026	63	-1	TCG			71.4
tRNA^Asn^	F	6029–6094	66	2	GTT			74.2
tRNA^Ser(AGN)^	F	6095–6162	68	0	GCT			71.5
tRNA^Glu^	F	6163–6227	65	0	TTC			86.2
tRNA^Phe^	R	6246–6311	66	18	AAA			72.8
*ND5*	R	6312–8031	1720	0		ATT	T	75.1
tRNA^His^	R	8047–8112	66	15	GTG			80.3
*ND4*	R	8113–9451	1339	0		ATG	T	76.6
*ND4L*	R	9453–9743	291	1		ATG	TAA	80.7
tRNA^Thr^	F	9746–9810	65	2	TGT			81.5
tRNA^Pro^	R	9811–9876	66	0	TGG			81.8
*ND6*	F	9879–10403	525	2		ATT	TAA	82.5
*CYTB*	F	10403–11539	1137	-1		ATG	TAG	69.1
tRNA^Ser(UCN)^	F	11538–11604	67	-2	TGA			77.6
*ND1*	R	11622–12569	948	17		TTG	TAA	75.5
tRNA^Leu(CUN)^	R	12571–12635	65	1	TAG			81.5
lrRNA	R	12636–13956	1321	0				80.6
tRNA^Val^	R	13957–14028	72	0	TAC			77.8
srRNA	R	14029–14811	783	0				77.6
Control region	–	14812–15878	1067	0				86.4

Note:

* IGN: Intergenic nucleotide, minus indicates overlapping between genes. tRNA^X^: where X is the abbreviation of the corresponding amino acid.

Among all sequenced cyclorrhaphan flies, the longest overlapping sequence was 17 bp, between *ND4* and *ND4L*, found in *Bactrocera minax* (Tephritidae) [20]. The longest intergenic sequence was located between *ND1* and *tRNA* ^*Ser(UCN)*^, (102 bp) in *Hypoderma lineatum* (Oestridae) [35]. There were four PCG–PCG gene boundaries in every cyclorrhaphan mt genome as in the ancestral insect mt genome: *ATP8*-*ATP6*, *ATP6*-*CO3*, *ND4L*-*ND4* and *ND6*-*CYTB*. *ATP8*-*ATP6* consistently overlapped by 7 bp with a-1 frame shift (A TGA TAA → ATG ATA A). Similarly, *ND4L*-*ND4* usually had a 7 bp overlap, except in the Opomyzoidea, Drosophilidae, and *Musca domestica* (Muscidae) [31] where these genes overlap by a single base A, in *Bactrocera minax* (Tephritidae) [20] there is a 17 bp overlap, and in *Megaselia scalaris* (Phoridae) (NC_023794) an 10 bp overlap. *N*. *mamaevi* is unique in that there is actually a single intergenic base between *ND4* and *ND4L*. *ATP6*-*CO3* often overlap by a single base, but is variable across cyclorrhaphan flies ranging from abutting genes without a gap or overlap (*Liriomyza sativae*, Agromyzidae [16] and *Liriomyza trifolii*, Agromyzidae [17]) to an intergenic space of up to 52 bp (*Drosophila virilis*, Drosophilidae [24]). Similarly, *ND6*-*CYTB* often overlap by a single base, but is variable across cyclorrhaphan flies ranging from an overlap of up to 8 bp (*Fergusonina taylori*, Fergusoninidae [14]) to intergenic sequences of up to 4 bp (*Liriomyza sativae*, Agromyzidae [16]; *Liriomyza trifolii*, Agromyzidae [17]; *Drosophila ananassae*, Drosophilidae [24]; *Stomoxys calcitrans*, Muscidae [32]).

### Base composition and codon usage

**Fig 2:**
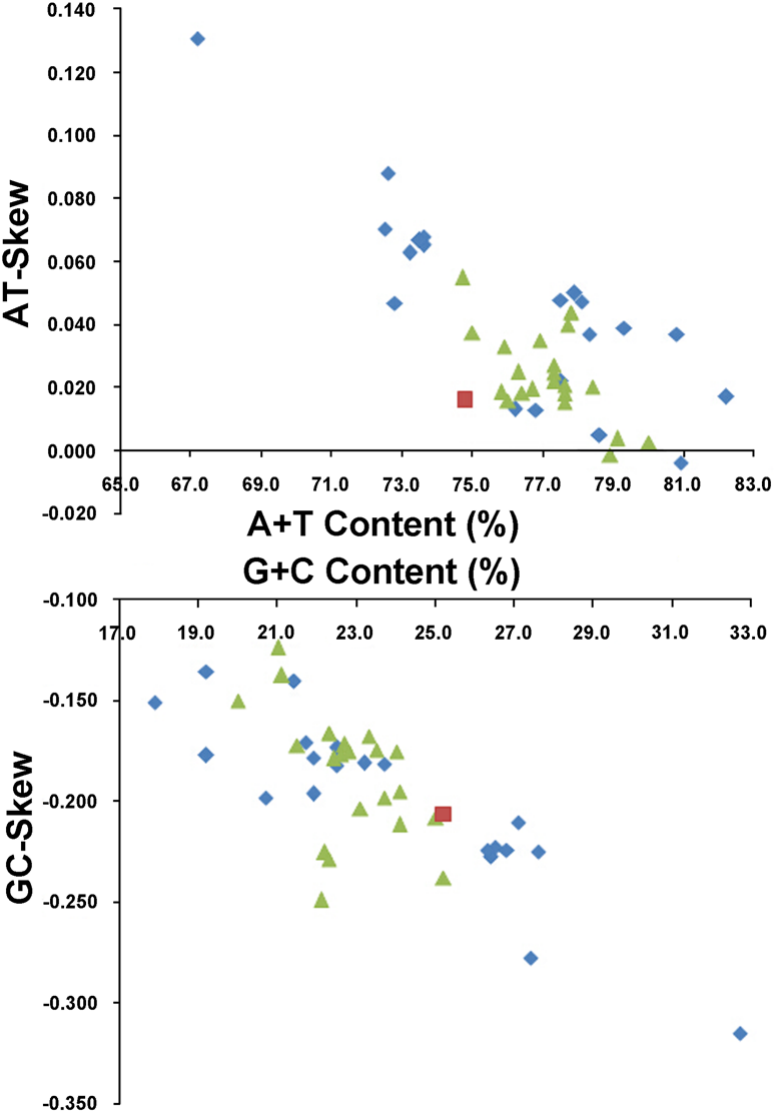
AT% vs AT-Skew and GC% vs GC-Skew in Cyclorrhapha mt genomes. Measured in bp percentage (X-axis) and level of nucleotide skew (Y-axis). Values are calculated on full length mt genomes. Blue rhombus, Acalyptratae; red square, Calyptratae; green triangle, *Nemopoda mamaevi*.

Similar to mt genome of other sequenced cyclorrhaphan flies, and indeed most insects, the nucleotide composition of the *N*. *mamaevi* was biased toward A and T (A = 38.0%, T = 36.8%, G = 10.0%, C = 15.2%; Table 3). The overall A+T content of the mt genome was 74.8%, average amongst all reported cyclorrhaphan flies, which range from 67.2% (in *Bactrocera minax*, Tephritidae [20]) to 82.2% (in *Drosophila melanogaster*, Drosophilidae [26]). A comparative analysis of nucleotide composition (A+T%, G+C%) versus skew (AT- and GC-skew) across the Cyclorrhapha is shown in Fig 2. The average AT-skew of the cyclorrhaphan mt genomes was 0.033, ranging from -0.004 (*Simosyrphus grandicornis*, Syrphidae [13]) to 0.131 (*Bactrocera minax*, Tephritidae [20]), whereas the *N*. *mamaevi* mt genome exhibits a weak AT-skew (0.016) (S3 Table). The average GC-skew of cyclorrhaphan mt genomes was -0.190, ranging from -0.315 (in *Bactrocera minax*, Tephritidae [20]) to -0.124 (in *Haematobia irritans*, Muscidae, NC_007102), while the *N*. *mamaevi* mt genome exhibited a marked GC-skew (-0.206) (S3 Table). AT- and GC- skews of Cyclorrhapha mt genomes are consistent with the strand skew biases found in most metazoan mt genomes (weakly positive AT-skew and strongly negative GC-skew for the J-strand). Within insects the only exceptions are found in three families: Philopteridae (Phthiraptera), Aleyrodidae (Hemiptera) and Braconidae (Hymenoptera), which have positive GC-skew and negative AT-skew on the J-strand [60] and in termites (Isoptera) which have strongly positive AT-skew on the J-strand [61]. In insects, the degree of AT-skew is related to gene direction, replication and codon positions, whereas the degree of GC-skew is affected by reversals in replication orientation [60].

**Table 3:** Nucleotide composition of the *Nemopoda mamaevi* mt genome.

Feature	A%	T%	C%	G%	A+T%	C+G%	AT-skew	GC-skew
Whole mitgenome	38.0	36.8	15.2	10.0	74.8	25.2	0.16	-0.21
PCGs	30.4	42.3	13.5	13.8	72.7	27.3	-0.16	0.01
1st codon position	30.8	36.0	12.7	20.5	66.8	33.2	-0.08	0.23
2nd codon position	20.4	46.0	19.5	14.2	66.4	33.7	-0.39	-0.16
3rd codon position	40.1	45.0	8.3	6.7	85.1	15.0	-0.06	-0.11
tRNA genes	38.2	37.9	10.7	13.2	76.1	23.9	0.00	0.10
lrRNA	39.1	41.5	6.4	12.9	80.6	19.3	-0.03	0.34
srRNA	37.9	39.7	7.8	14.6	77.6	22.4	-0.02	0.30
Control region	44.5	41.9	9.1	4.5	86.4	13.6	0.03	-0.05

Note: The A+T and G+C biases of protein-coding genes were calculated by AT-skew = [A-T]/[A+T] and GC-skew = [G-C]/[G+C], respectively.

**Fig 3:**
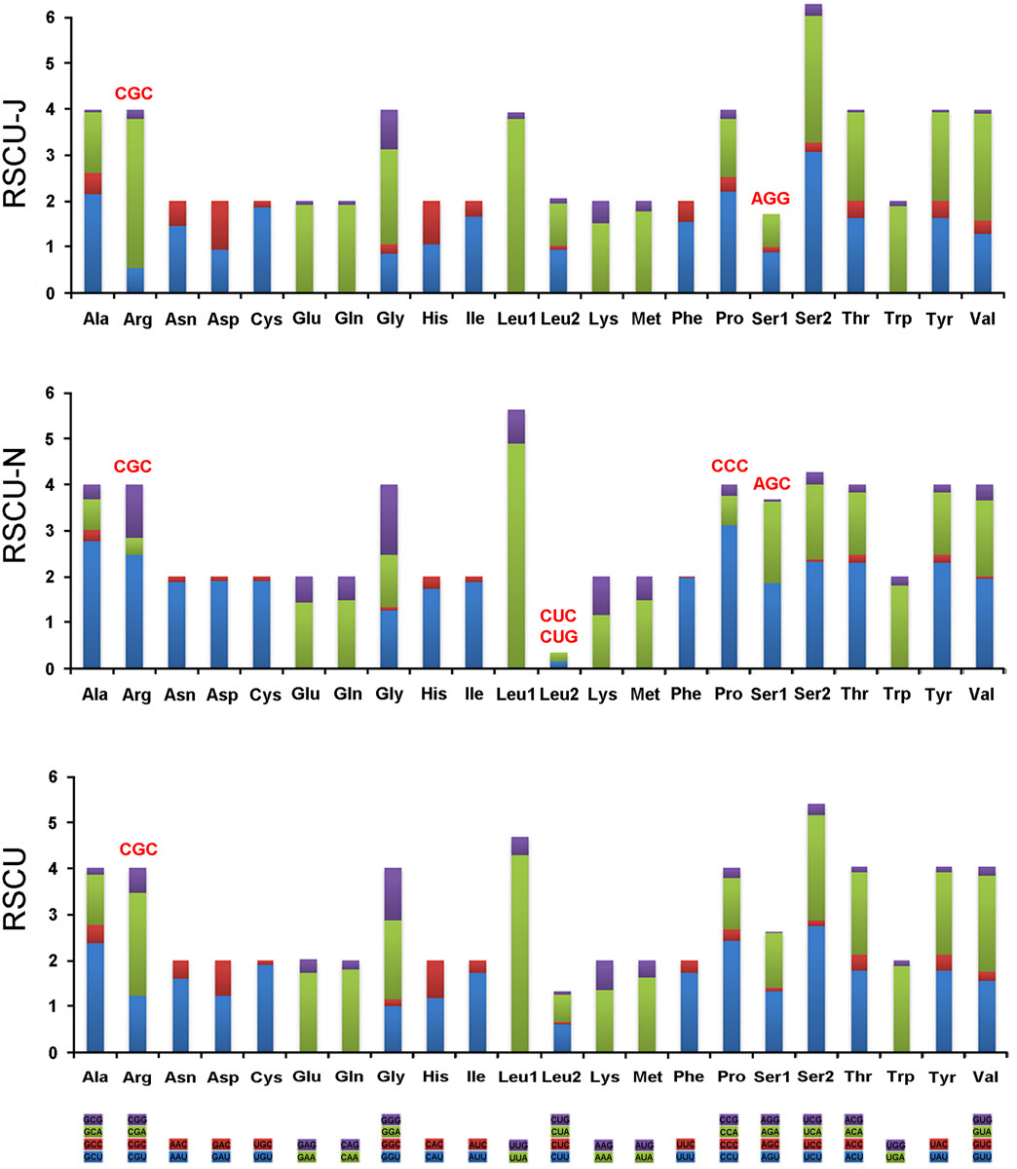
Relative synonymous codon usage (RSCU) in the *Nemopoda mamaevi* mt genome. Codon families are provided on the X-axis. Stop codon is not given. Red codon, codon not present in the chain/genome.

A and T bias is also reflected in relative codon usage by the PCGs. In the mt genome of *N*. *mamaevi*, A+T rich codons, such as ATT (Asn), TAA (Leu), AAA (Lys), ATA (Met), TTT (Phe), TAT (Tyr), are more frequently used than G+C rich codons. Among both strands, NNA-form codons were the most frequently used and NNC-type the most infrequently used codons. On J-strand NNA were preponderant and NNG were the least frequent with the reverse found in N-strand encoded PCGs (NNU commonest, NNC least common) (Fig 3, S4 Table).

### Protein-coding Genes

The overall A+T content of the 13 PCGs in *N*. *mamaevi* was 72.7%, with individual PCGs ranging from 65.8% (*CO1*) to 82.5% (*ND6*) (Table 2). The A+T content of the third codon positions (85.1%) were much higher than either the first (66.8%) or second codon (66.4%). There was moderate negative AT-skew for the PCGs as a whole (-0.16) driven by strongly negative AT-skew at second codon positions (-0.39), with weak skew at first, and third codon positions (-0.08 and -0.06 respectively). The absence of significant GC-skew across the PCGs as a whole (0.01) masks strong skews at each codon position with first codon positions strongly positive (0.23) balanced by strongly negative skews at second and third codon positions (-0.16 and -0.11 respecively) (Table 3).

ATN, GTG (Val) and TTG (Leu^UUR^) are accepted as the canonical start codons for invertebrate PCGs [61], while TCG (Ser^UCN^) has been proposed to be an additional start codon commonly found in flies [13]. All 13 PCGs in the *N*. *mamaevi* mt genome used canonical start codons. *ND2*, *ND5* and *ND6* started with ATT (Ile); ATC (Ile) were found in *ATP8* and *ND3*; *CO1* and *ND1* start with TCG (Ser^UCN^) and TTG (Leu^UUR^), respectively; and ATG (Met) were used in the remaining six PCGs as start codons (Table 2).

**Fig 4:**
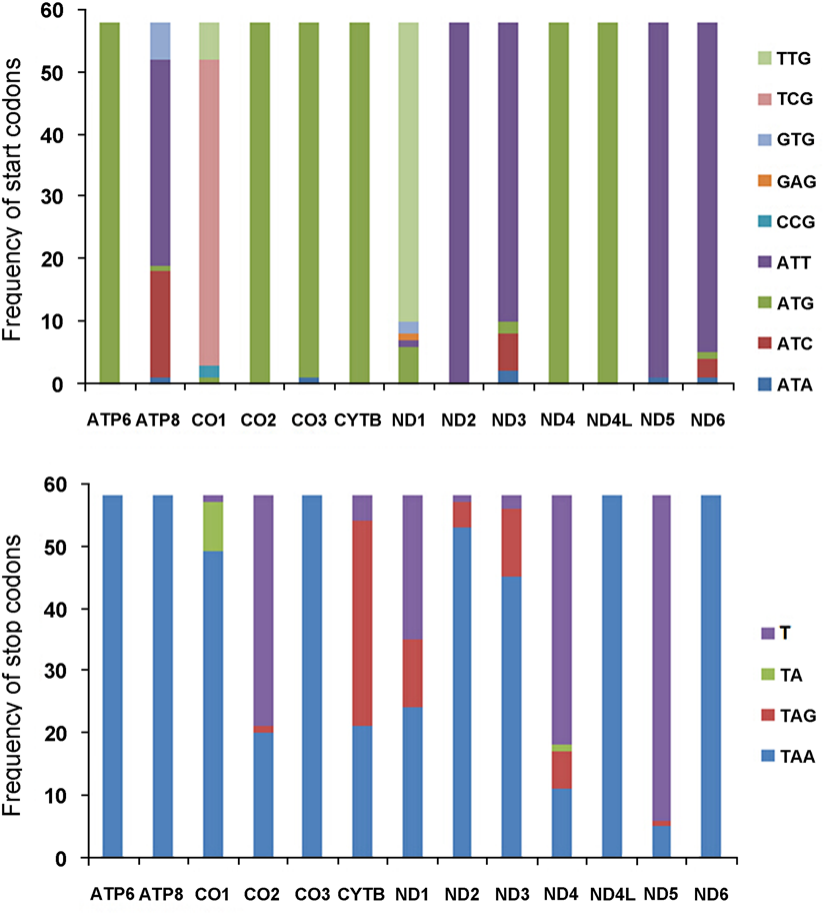
Usage of start and stop codons in complete Cyclorrhapha mt genomes. (A) Start codons usage of 13 PCGs in Cyclorrhapha. (B) Stop codons usage of 13 PCGs in Cyclorrhapha.

Among the cyclorrhaphan flies sequenced to date, ATG (Met) is the most frequently used start codon followed by ATT (Ile). ATG (Met) is almost exclusively used in the *ATP6*, *CO2*, *CO3*, *CYTB*, *ND4* and *ND4L* genes of almost all cyclorrhaphan species (*Procecidochares utilis*, Tephritidae, NC_020463) which uses ATA (Met) for *CO3*). ATT (Ile) is used for *ND2* in all species as well as *ATP8*, *ND3*, *ND5* and *ND6* in most species. Other standard start codons such as ATA (Met) and ATC (Ile) are also found in *ATP8*, *CO3*, *ND3*, *ND5* and *ND6* in a minority of fly species. TTG (Leu^UUR^) is used in *ND1* in most species and TCG (Ser^UCN^) is used in *CO1* in most species. (Fig 4, S5 Table). In addition, three non-canonical start codons GTG (Val), CCG (Pro) and GAG (Glu) have been proposed. GTG (Val) is found as the start codon for *ATP8* in six *Bactrocera* species (Tephrididae) and for *ND1* in two *Liriomyza* flies (Agromyzidae). CCG (Pro) is in the start codon of *CO1* in *Drosophila sechellia* and *D*. *simulans* (Drosophilidae) [29]. GAG (Glu) was proposed as the start codon for *ND1* in *Rutilia goerlingiana* (Tachinidae) [5]. *ATP8*, *CO1*, *ND1*, *ND3* and *ND6* therefore collectively utilise three to five different start codons. In contrast, the remaing of the PCGs utilise no more than two and often only one, start codon (Fig 4, S5 Table).

The stop codons most commonly used in *N*. *mamaevi* are TAA (*ATP6*, *ATP8*, *CO1*, *CO2*, *CO3*, *ND1*, *ND2*, *ND4L*, *ND6*), or TAG (*CYTB*, *ND3*) found in 11 of the 13 PCGs. The remaining two PCGs (*ND4*, *ND5*) utilise the partial stop codon T, which has been found in many insect mt genomes and is completed to a full TAA stop codon via post-transcriptional polyadenylation [63]. In all reported cyclorrhaphan flies studied to date, TAA is the most common stop codon used found in every PCG in at least one species, most notably in the *ATP6*, *ATP8*, *CO3*, *ND4*, *ND4L* and *ND6* genes from all species sequenced. TAG has been found in *CO2*, *CYTB*, *ND1*, *ND2*, *ND3*, *ND4* and *ND5*. The incomplete stop codons TA and T are founded in *CO2*, *CYTB*, *ND1*, *ND2*, *ND3*, *ND4* and *ND5* as well as being commonly found in *CO1* (Fig 4, S5 Table).

By aligning the homologous genes PCGs among closely related species, we detected an apparent sequencing error in the published mt genomes of *Bactrocera minax* (Tephritidae) [20]. Base position 896 bp of *ND1* in *B*. *minax*, a C, is apparently an insertion as it results in a frameshift in the downstream amion-acid sequence that is significantly different from other cyclorrhaphan flies; removal of this C restores the correct reading frame resulting in the same amino acid sequence as in other cyclorrhaphans and the same stop-codon. In addition, *CO2* in *Procecidochares utilis* (Tephritidae) (NC_020463) has an apparent deletion at position 678, resulting in a missing “A” base. As a result, the version of this genome on GenBank is 5 bp too long at the 3’ end.

### Intergenic sequences

Two conserved intergenic sequences blocks of genes coded on different strands (7 bp between *ND1* and *tRNA* ^*Ser(UCN)*^, 5 bp between *tRNA* ^*Glu*^ and *tRNA* ^*Phe*^) were found in typical insect mt genome [64–65]. Here, we revealed two 18 bp conserved sequences from both spacers across Cyclorrhapha.

**Fig 5:**
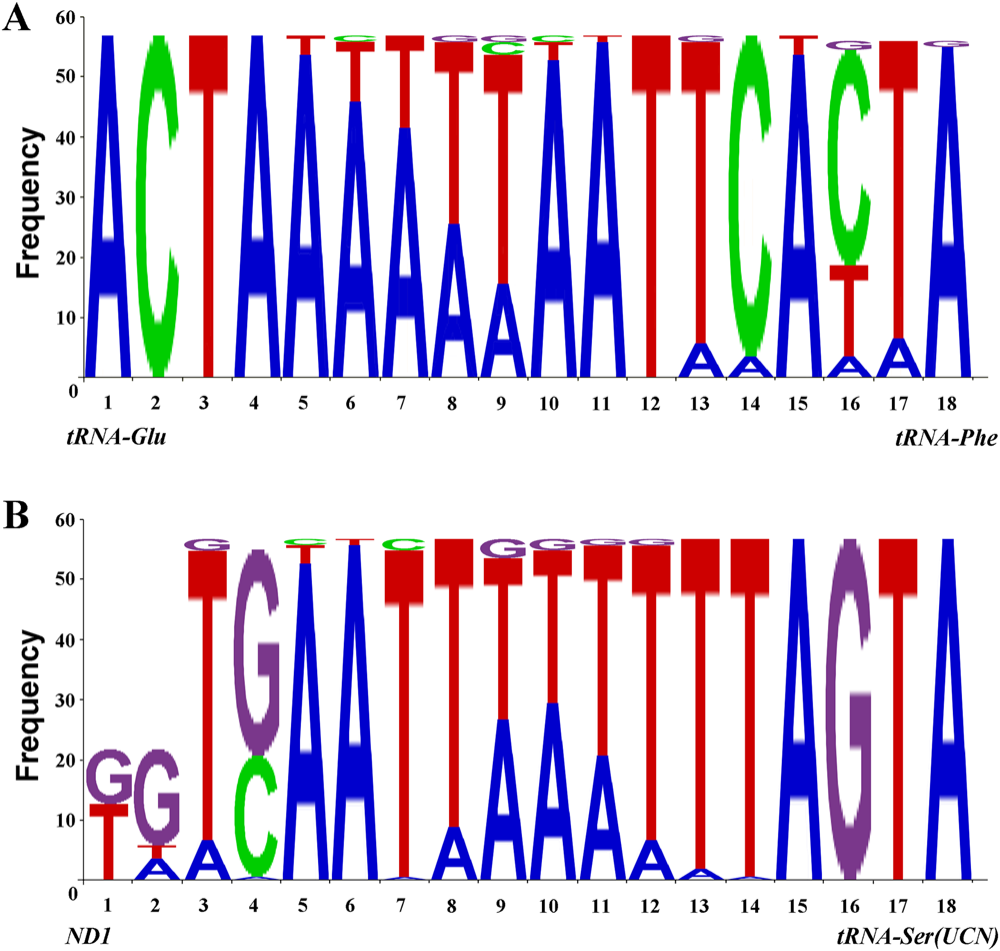
Two conserved intergenic sequences. (A) Sequences between *tRNA* ^*Glu*^ and *tRNA* ^*Phe*^, forward sequences. (B) Sequences between ND1 and tRNA^Ser(UCN)^, reversed sequences.

The non-coding region between *tRNA* ^*Glu*^ and *tRNA* ^*Phe*^ is found in all Cyclorrhapha, although ranging from 65 bp (*Fergusonina taylori*, Fergusoninidae [14]) to 16 bp (*Sarcophaga peregrine*, Sarcophagidae, NC_023794), often 18 bp in length (except 17 bp in *Hypoderma lineatum*, Oestridae [35]; 19 bp in *Drosophila grimshawi*, Drosophilidae [24]; 20 bp in *Drosophila mojavensis*, Drosophilidae [25] and 23 bp in *Simosyrphus grandicorni*, Syrphidae [13], respectively). Sequences from 57 species (*Pollenia rudis* was excluded because its intergenic sequences were replaced by an 18 bp TATA box) were analysed and presented in Fig 5A.

The non-coding region located between *ND1* and *tRNA* ^*Ser(UCN)*^ is the binding site of mtTERM, a transcription termination peptide which has a highly conserved 7-bp motif across insects [66]. MtTERM functions to control over-expression of the rRNA genes relative to the protein-coding genes [64, 67].

The annotated sequence length and stop codons of *ND1* varies considerably among sequences from cyclorrhaphan flies. For example all Calyptratae sequenced to date have canonical stop codons (TAA or TAG). Most of the sequenced Acalyptratae species lack complete stop codons, resulting in significant length variability. Moreover, some species lack even incomplete stop codons e.g. the bases immediately preceding *tRNA* ^*Ser(UCN)*^ are neither T nor TA. We aligned the region from *ND1* to *tRNA* ^*Ser(UCN)*^ of all sequenced Cyclorrhapha, and re-annotated *ND1* gene based on relatively well conserved sequences in this intergenic spacer and despite large variation in spacer length (e.g. up to 102 bp *Hypoderma lineatum*, Oestridae [35]). Sequences from 57 species/subspecies populations were aligned and analysed (excluding *Bactrocera minax* due to the sequencing errors noted above). Although the intergenic sequences between *ND1* and *tRNA* ^*Ser(UCN)*^ ranges from 15–102 bp in size, there is a 16 bp highly conserved sequence, found in all Cyclorrhapha (except *Drosophila simulans* and *Rutilia goerlingiana* in which there is a 1 bp deletion). Species that lack complete stop codons in *ND1* have two additional, poorly conserved, bases at the 5’ end of this 16 bp conserved region. Nucleotide usage frequency at each position in this conserved region is shown in Fig 5B.

### Transfer RNAs

**Fig 6:**
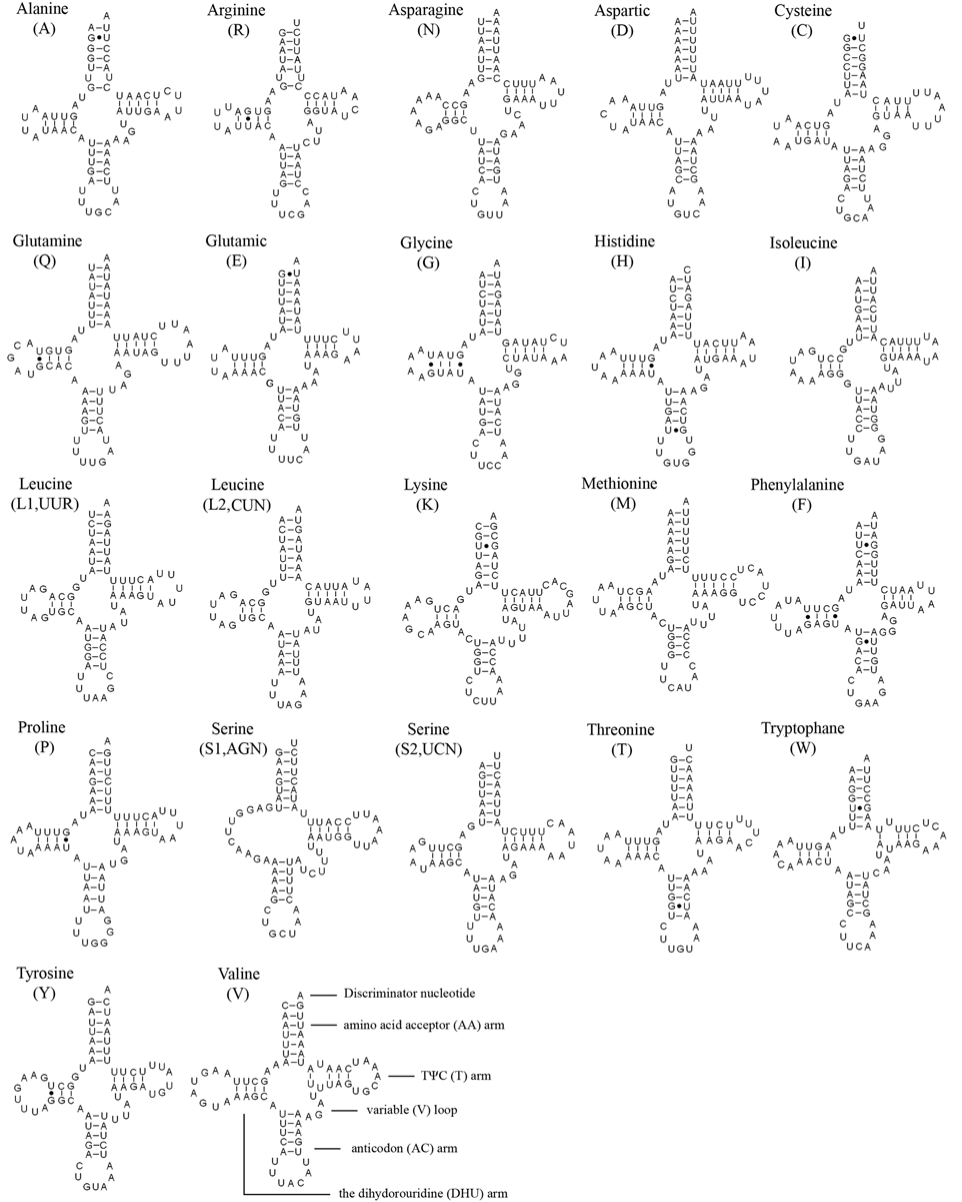
Putative secondary structures of tRNAs found in the mitochondrial genome of *Nemopoda mamaevi*. All tRNAs can be folded into the usual clover-leaf secondary structure. The tRNAs are labelled with the abbreviations of their corresponding amino acids. Inferred Watson-Crick bonds are illustrated by lines, whereas GU bonds are illustrated by dots.

The typical complement of 22 typical tRNAs found in the arthropod mt genomes were found in the *N*. *mamaevi* mt genome, ranging in size from 63 bp (*tRNA* ^*Arg*^) to 72 bp (*tRNA* ^*Val*^) and 1465 bp or 9.23% of the total genome. The overall A+T content the tRNAs was 76.2%, while single genes ranged from 64.8% (*tRNA* ^*Lys*^) to 86.6% (*tRNA* ^*Asp*^). Fourteen genes are encoded on the J-strand and the remains are encoded on the N-strand. Most tRNAs could be folded into the typical clover-leaf structure (Fig 6), whereas the *tRNA* ^*Ser(AGN)*^ was an exception lacking a DHU arm as has been observed for this gene in other metazoan mt genomes [62].

A total of 17 G–U pairs and two mismatched base U–U pairs were found in *N*. *mamaevi* mitochondrial tRNA secondary structures, no A–A pairs or A–C pairs were found. The G–U pairs are located in the AA arm (7 bp), DHU arm (8 bp), AC arm (2 bp), respectively. In contrast, all mismatched base U–U pairs are located in the TψC arm.

### Ribosomal RNAs

The rRNAs of *N*. *mamaevi* were 1,321 bp for *lrRNA* and 783 bp for *srRNA* in length. Their A+T content were 80.6% and 77.6%, respectively. Because rRNA genes lack functional annotation features, analogous to the start and stop codons of PCGs, it is impossible to determine the boundaries from DNA sequence alone [11, 68]. Hence, they were assumed to extend to the boundaries of flanking genes. As in other dipteran species, the *lrRNA* gene is flanked by *tRNA* ^*Leu (CUN)*^ and *tRNA* ^*Val*^, while the *srRNA* gene is flanked by *tRNA* ^*Val*^ and the control region.

**Fig 7:**
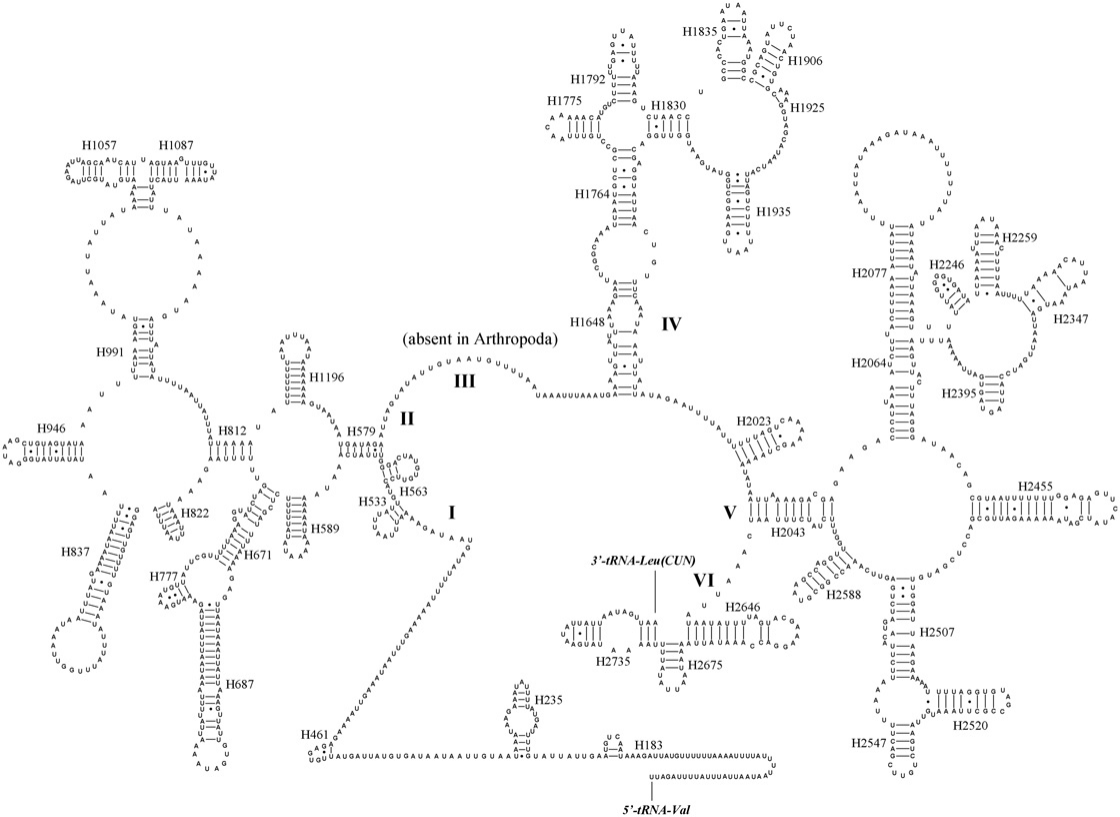
Predicted secondary structure of the *lrRNA* gene in *Nemopoda mamaevi*. Inferred Watson–Crick bonds are illustrated by lines, GU bonds by dots.

**Fig 8:**
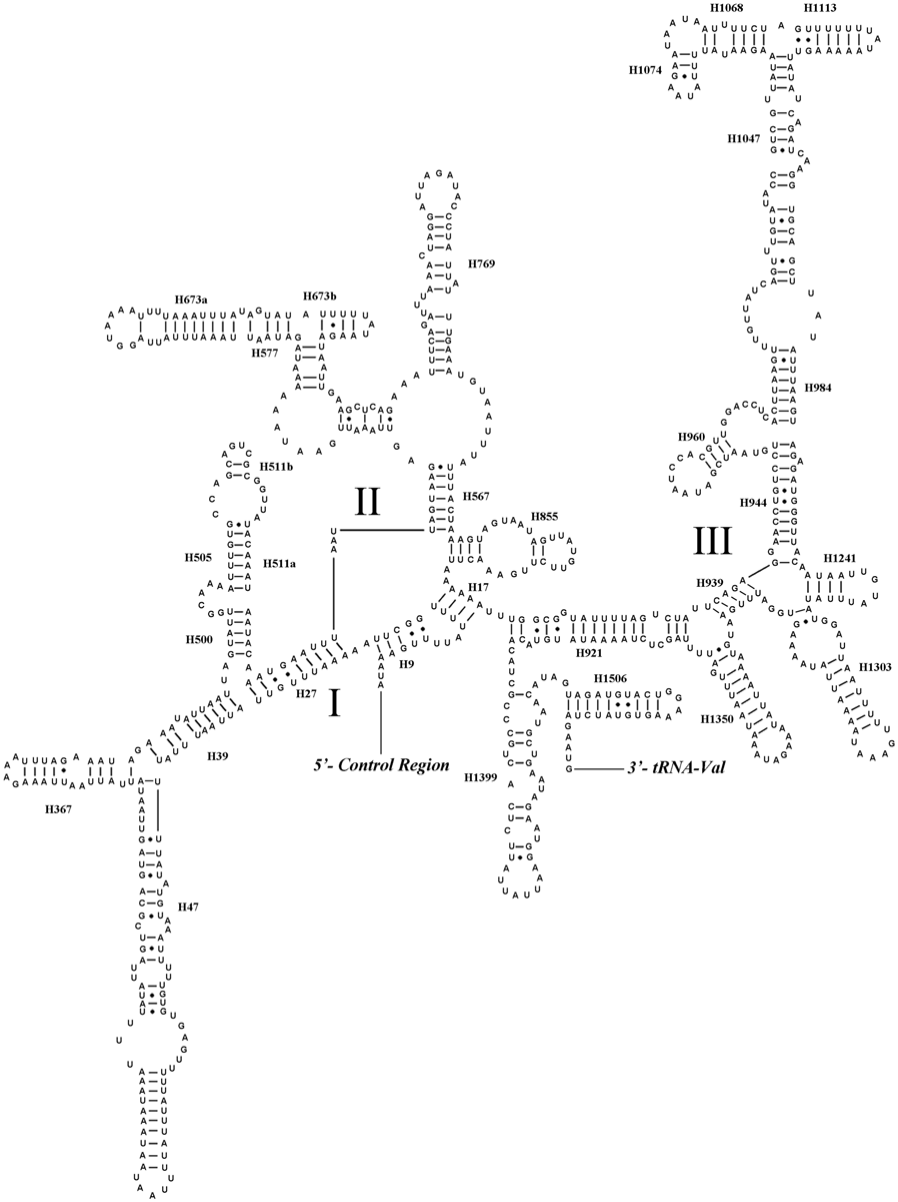
Predicted secondary structure of the *srRNA* gene in *Nemopoda mamaevi*. Roman numerals denote the conserved domain structure. Inferred Watson-Crick bonds are illustrated by lines, GU bonds by dots.

We inferred secondary structures of *lrRNA* and *srRNA* of *N*. *mamaevi* using the published rRNA secondary structures of a leafminer *Liriomyza sativae* (Agromyzidae), the only dipteran so analysed to date, as a model [16]. The *lrRNA* had 49 helices in five structural domains (I-II, IV-VI, domain III is absent as in other insects), similar to *L*. *sativae* [16] and typical of arthropods [69]. The secondary structures of *srRNA* in *N*. *mamaevi* included three domains and 33 helices, again similar to other Diptera [16] (Figs 7 and 8).

### The control region

**Fig 9:**
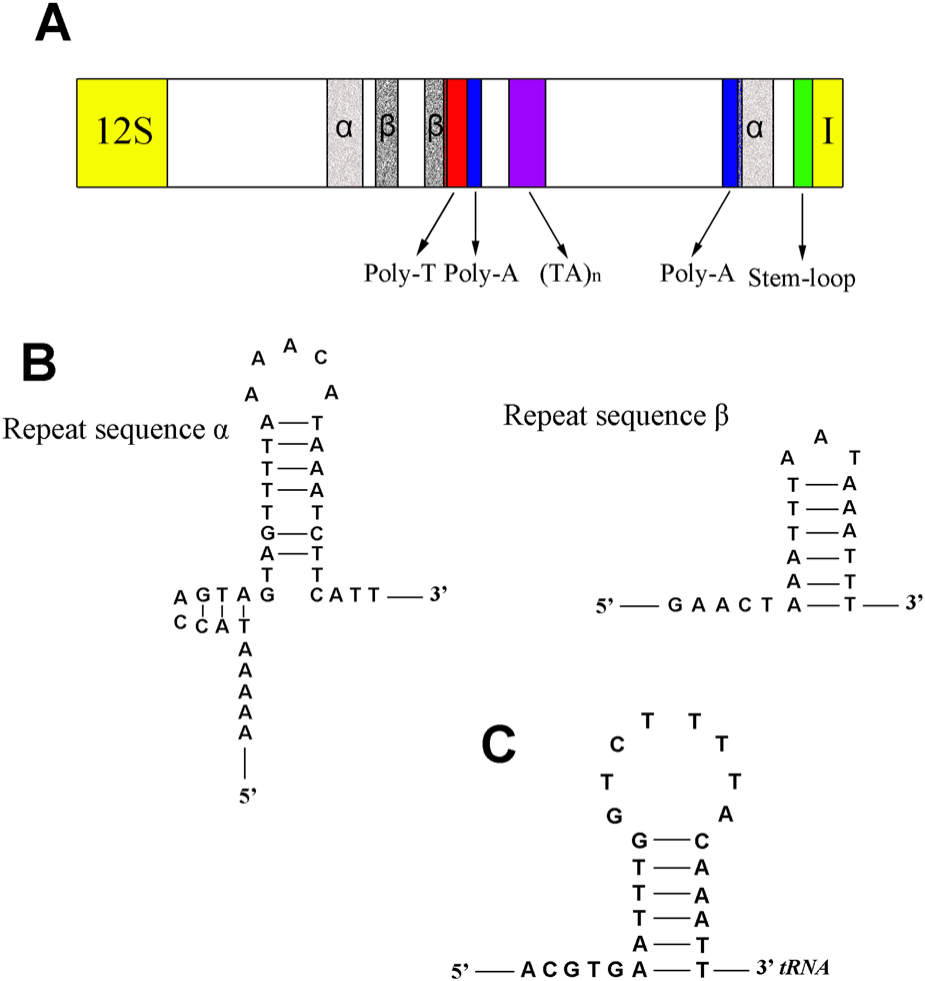
Predicted structure elements in the control region of *Nemopoda mamaevi*. A. control region structure of *Nemopoda mamaevi*, B. secondary structures of repeat sequences of *Nemopoda mamaevi*, C. secondary structures of stem-loop structure at 3’-end.

The control region of *N*. *mamaevi* is 1067 bp long and located at the conserved insect position between *srRNA* and *tRNA* ^*Ile*^. Control region A+T content was 86.4%, second only to *tRNA* ^*Asp*^ (86.6%) as the highest A+T content region in the *N*. *mamaevi* genome. Of the five conserved structural elements identified by Zhang and Hewitt (1997) [70] across insect mt control regions, we could detect three of them: (1) a 21 bp of poly-T stretch, which may be associated with the control of transcription and replication (Fig 9A); (2) a (TA)_n_ like stretch; (3) a stem-loop structure at 3’-end of control region, however the 5’ ‘TATA’ and a 3’ ‘G(A)_n_T’ consensus regions were apparently absent (Fig 9C). We identified two additional, structural elements, not conserved in other insects and apparently unique to *N*. *mamaevi*: (1) two poly-A stretches (10 bp and 22 bp in length), (Fig 9A); and (2) two non-tandem macro repeats: 5’-AAAAAATACCAGTAGCTGTTTTAAAACATAAATCTTCATT-3’ (from 313 to 352, from 993 to 1,032), and 5’-GAACTAAATTTAATAAAATTT-3’ (from 372 to 392, from 498 to 518). Both macro repeats could be folded into stem-loop structures (Fig 9B) and the second unit of each repeat type overlaps the poly-A and poly-T stretches, respectively (Fig 9A).

### Automated annotation method with MITOS

MITOS (http://mitos.bioinf.uni-leipzig.de/index.py) [49] is the most advanced automated mt genome annotation pipeline yet produced, and is able to be operated over an external server allowing the batched submission of mt genomes without tying up the user’s computer. However the accuracy of its annotations of protein-coding genes, in particular the location of start and stop codons, has been criticised [3]. We used MITOS to the mt genome of *N*. *mamaevi*, and compared the results with our hand annotation results.

MITOS identified all 37 genes in the correct gene order and direction on the mt genome. However not all the PCGs were correctly annotated, only the start codons of *ATP8*, *CO2*, *CO3*, *CYTB*, *ND6* and stop codons of *ND1*, *ND4*, *ND4L*, *ND5* were correct (S6 Table). For the tRNA genes, there was only one incorrect annotation: *tRNA* ^*Ala*^ lacked a base A at 3’-end (S6 Table). In contrast an older, widely used tRNA prediction package tRNAscan-SE Search Server v.1.21 (http://lowelab.ucsc.edu/tRNAscan-SE/) [46] proposed 25 potential tRNAs, and while 20 were correct, *tRNA* ^*Arg*^ and *tRNA* ^*Ser(AGN)*^ were not detected, and three spurious tRNA genes were predicted (S7 Table). These results are consistent with previous comparisons in the order Lepidoptera, that found MITOS outperformed tRNAScan for tRNA prediction, but was inferior to hand annotation of PCGs [3].

### Phylogeny

Four datasets were used in the phylogenetic analysis, there are 11,058 residues in the PCG123 matrix (containing nucleotides of 13 PCGs), 14,226 residues in the PCG123RNA matrix (containing nucleotides of 13 PCGs, two rRNAs and 22 tRNAs), 7,372 residues in the PCG12 matrix (containing nucleotides of 13 PCGs but excluding the third codon sites) and 10,540 residues in the PCG12RNA matrix (containing nucleotides of 13 PCGs but excluding the third codon sites, two rRNAs and 22 tRNAs).

**Fig 10:**
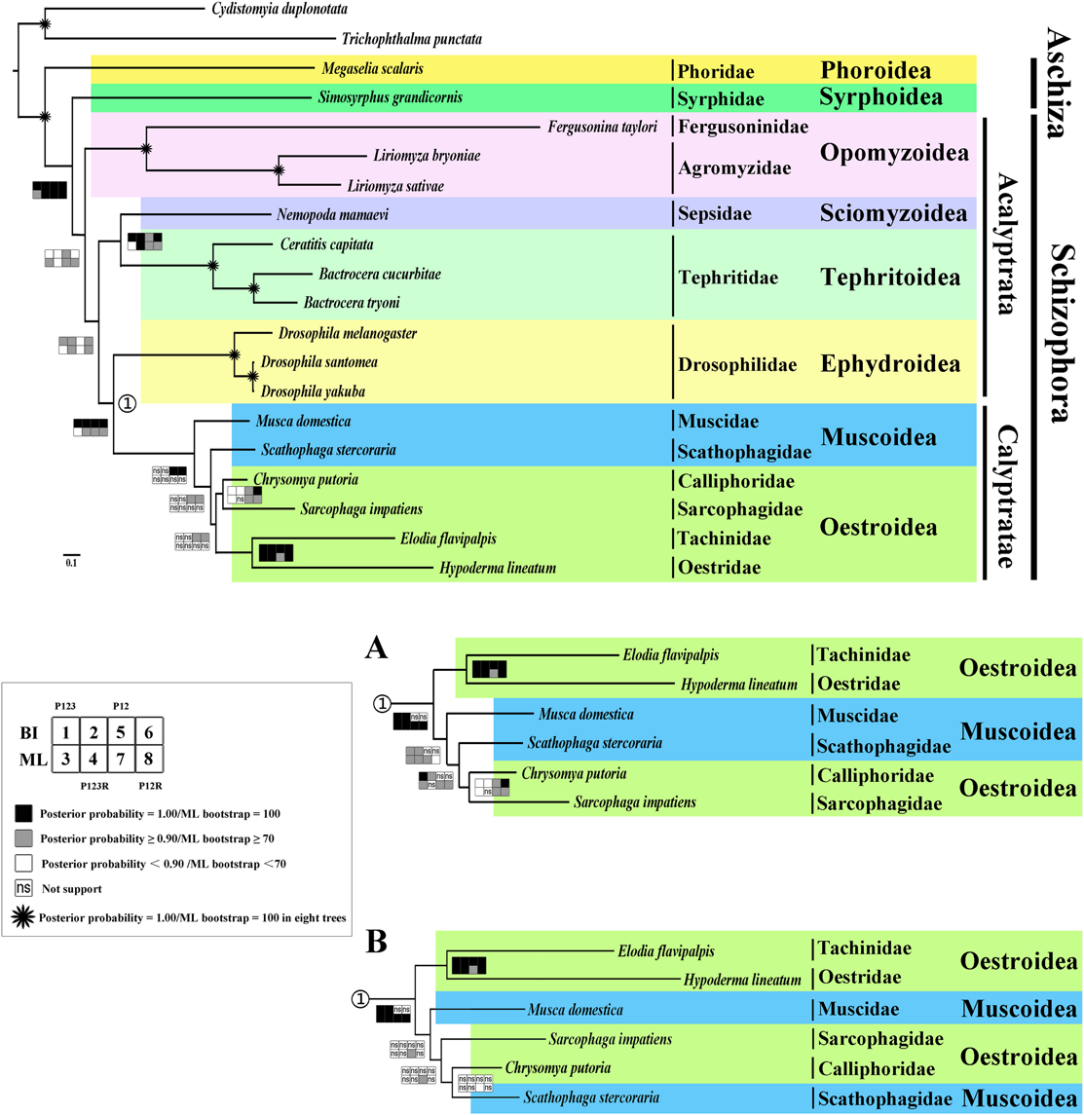
Phylogenetic tree of Brachycera families based on mt genome data. Cladogram of relationships resulting from Bayesian analyses with datasets PCG12 and PCG12RNA, with *Cydistomyia duplonotata* (Nemestrinidae) and *Trichophthalma punctata* (Tabanidae) as outgroups. Squares at the nodes are Bayesian posterior probabilities for 1, 2, 5 and 6, ML bootstrap values for 3, 4, 7 and 8. Dataset of PCG123, 1 and 3, PCG123RNA, 2 and 4, PCG12, 5 and 7, PCG12RNA, 6 and 8. Black indicates posterior probabilities = 1.00 or ML bootstrap = 100, gray indicates posterior probabilities ≥ 0.90 or ML bootstrap ≥ 70, white indicates posterior probabilities < 0.90 or ML bootstrap < 70, ‘ns’ indicates not support, * indicates posterior probabilities = 1.00 or ML bootstrap = 100 in eight trees. A. Part of the Bayesian tree of datasets PCG123 and PCG123RNA as well as ML tree of datasets PCG123, PCG12 and PCG12RNA. B. Part of the ML tree of dataset PCG123RNA.

The phylogenetic trees conducted from both Bayesian and ML analyses have very similar topologies across four datasets (Fig 10). The monophyly of Cyclorrhapha, Opomyzoidea, Tephritoidea and Ephydroidea were consistently supported (posterior probability = 1.00, ML bootstrap = 100), as was the monophyly of Schizophora (posterior probability = 1.00, ML bootstrap = 85/100/100/100) and Calyptratae (posterior probability = 1.00, ML bootstrap = 41/73/87/84). Several recent researchers have concluded that ‘Aschiza’ is not monophyletic [71–76], here we add support for this conclusion from mt genome data. Phoridae was sister group of other Cyclorrhapha (posterior probability = 1.00, ML bootstrap = 100), and Syrphoidea was sister group of the Schizophora (posterior probability = 1.00, ML bootstrap = 85/100/100/100). This supports Wiegmann et. al’s findings regarding basal branching events within the Cyclorrhapha [77], however their nodal support for the relationship Phoridae + (remaining Cyclorrhapha) was weaker than that found here. Although only moderately supported (posterior probability = 0.86/0.70/0.98/0.79, ML bootstrap = 78/14/95/82), the position of superfamily Opomyzoidea (Fergusoninidae + Agromyzidae) was sister to the remaining Cyclorrhapha. Wiegmann et al. [77] did not find a monophyletic Opomyzoidea, nor did they find a sister-group relationship between the two representative families included in the present study. Given that there are 14 families recognized within the Opomyzoidea considerable additional data is necessary to firmly resolve their monophyly and relationships to other schizophoran superfamilies. Sepsidae is sister taxon to Tephritoidea (posterior probability = 1.00/1.00/0.92/1.00, ML bootstrap = 33/100/91/99), while Ephydroidea + Calyptratae formed a monophyletic group (posterior probability = 1.00, ML bootstrap = 41/73/87/84). Both relationships have previously been found in Wiegmann et al. [77], however neither had significant nodal support in that study.

The primary difference among the eight phylogenetic trees here is in the relationships of the superfamily Muscoidea (represented by the families Muscidae and Scathophagidae) and Oestroidea (represented by the families Oestridae, Tachinidae, Calliphoridae and Sarcophagidae). In the Bayesian analysis of datasets PCG12RNA and PCG12, the monophyly of Oestroidea was supported (posterior probability = 0.99/0.99), while a paraphyletic Muscoidea formed a grade at base of Calyptratae. Our results provide further evidence that ‘Muscoidea’ is paraphyletic as found in previous studies [77–78]. The Oestroidea was only monophyletic for the datasets that removed third codon positions (PCG12RNA and PCG12) but only when analysed by Bayesian inference. For the datasets that included third codon positions, and all analyses using maximum likelihood as the inference method, Oestroidea was rendered paraphyletic by the muscoid families which formed a clade with Calliphoridae and Sarcophagidae. Two pairs of relationships within Oestroidea were consistently supported by our analyses: Calliphoridae + Sarcophagidae formed a monophyletic group in seven of the eight analyses (posterior probability = 0.70/0.84/0.99/1.00, ML bootstrap = 58/-/98/90), while Oestridae + Tachinidae was strongly supported in all eight analyses (posterior probability = 1.00/1.00/1.00/1.00, ML bootstrap = 100/100/98/100). One of three sets of relationships within Calyptratae were found depending on the combination of dataset and analysis methods: monophyletic Oestroidea (BI-PCG12, BI-PCG12RNA: Fig 10), Muscidae + (Scathophagidae + (Calliphoridae + Sarcophagidae)) (BI-PCG123, BI-PCG123RNA, ML-PCG123, ML-PCG12, ML-PCG12RNA: Fig 10A) or Muscidae + (Sarcophagidae + (Calliphoridae + Scathophagidae)) (ML-PCG123RNA: Fig 10B).

The paraphyly of Oestroidea as found here in the majority of analyses has not been supported in previous studies such as those by Wiegmann et al. [77] and Kutty et al. [78]. Both studies found a monophyletic Oestroidea but with differing relationships between families: Wiegmann et al. [77] inferred ((Oestridae + Sarcophagidae) + (Tachinidae + Calliphoridae)), while Kutty et al. [78] found the relationships (((Tachinidae + Oestridae) + Calliphoridae) + Sarcophagidae) with both Calliphoridae and Tachinidae paraphyletic (Oestridae is nested within Tachinidae, as are some calliphorid subfamilies).

Phylogenetic analyses of mitochondrial genome data are known to be susceptible to compositional bias, particularly of third codon positions in the protein-coding genes [2] particularly in the Diptera [5, 13], and it is likely that the results found here of unstable relationships within the Calyptratae and a paraphyletic Oestroidea are an example of this phenomena. Even if comparisons between the present study and the previous ones by Wiegmann et al. [77] and Kutty et al. [78] are restricted to comparing trees that find a monophyletic Oestroidea, there is considerable variation in inferred relationships with Oestridae + Tachinidae, the only interfamilial relationships found in more than one study (present study and [78]). Considerable additional data is required to reliably resolve relationships within the Oestroidea, while our results suggest the reliability of mt genome data in inferring phylogenetic relationships within the Cyclorrhapha generally, caution is warranted for its use within the Oestroidea given that we have demonstrated susceptibility to third codon biases.

## Supporting Information

S1 Table Primers used in this study. (DOCX)

S2 Table The best partitioning scheme selected by PartitionFinder for different dataset. (DOCX)

S3 Table AT%, GC%, AT-Skew and GC-Skew in complete Cyclorrhapha mt genomes. (DOCX)

S4 Table Codon usage of the *Nemopoda mamaevi* mt genome. (DOCX)

S5 Table Start and Stop codons of Cyclorrhapha mt genomes. (XLSX)

S6 Table Automated annotation results of the *Nemopoda mamaevi* mt genome by MITOS. (DOCX)

S7 Table Annotation results of the *Nemopoda mamaevi* tRNAs by tRNAscan-SE. (DOCX)
