# Supplementary material for: The First Mitochondrial Genome of the Sepsid Fly Nemopoda mamaevi Ozerov, 1997 (Diptera: Sciomyzoidea: Sepsidae), with Mitochondrial Genome Phylogeny of Cyclorrhapha
Source: PLoS One. 2015 Mar 31;10(3):e0123594. doi: 10.1371/journal.pone.0123594 (PMC4380458; doi:10.1371/journal.pone.0123594)
Supplement: S1 Table — (DOCX) [file pone.0123594.s001.docx]

**S1 Table.** Primers used in this study.

| Number | Primer pairs (F/R) | Sequence (forward and reverse) 5’-3’ | Size (bp) |
| --- | --- | --- | --- |
| 1 | TM-J-206/N2-N-732 | GCTAAATAAGCTAACAGGTTCAT/AAGGAAGTTTGGTTTAAACCTCC | 550 |
| 2* | NEM-J-530/NEM-N-1689 | CCTTTCCATTTTTGGTTTCC/GATATAGCTTTCCCCCGAATAAA | 1200 |
| 3 | TY-J-1460/C1-N-2191 | TACAATCTATCGCCTAAACTTCAGCC/CCCGGTAAAATTAAAATATAAACTTC | 700 |
| 4 | C1-J-1751/TL2-N-3014 | GGAGCTCCTGATATAGCATTCCC/TCCATTGCACTAATCTGCCATATTA | 1300 |
| 5 | Cl-J-2183/C3-N-5460 | CAACATTTATTTTGATTTTTTGG/TCAACAAAGTGTCAGTATCATGC | 3200 |
| 6 | C2-J-3530/A6-N-4493 | AAGTTGATGGAACTCCTGGACG/CTGTTAATCGAACTGCTAAAGTTC | 1000 |
| 7 | C3-J-5005/E-rev | CTCCAGCAATTGAATTAGGAGCTA/AGTGATAAGCCTCTTTTTGGCTTC | 1100 |
| 8 | F-fw/N5-N-7707 | CATTTGATTTGCATTCAAAAAGTATTG/AGGATGAGATGGATTAGGACTAG | 1700 |
| 9* | NEM-J-7441/ NEM-N-8619 | AAAAGGAATCTGAGCTCTCTTAGT/GAAGAATAAGCAATTAATGCCTT | 1200 |
| 10 | I-fw/N4-N-8924 | CTATTTAATAAAGAAATTTCTCC/CCTAAAGCTCATGTTGAAGCTCC | 450 |
| 11 | N4-J-8614/N4-N-9061 | TGAGCAACAGAAGAATAAGC/ATCAACCTGAACGATTACAAG | 400 |
| 12 | N4-J-8944/I-rev | CAGGAGCTTCAACATGAGCTTTAGG/CTTATTTTTGATTTACAAGACCAATG | 1000 |
| 13 | N4-J-9511/CB-N-11218 | CCAAAATTGATAACCCTAAAGC/TCAGGTTGAATGTGAATTGG | 1700 |
| 14 | CB-J-10933/N1-N-12051 | TATGTTCTACCATGAGGACAAATATC/GATTTTGCTGAAGGTGAATCAGA | 1200 |
| 15* | NEM-J-11891/NEM-N-12827 | AACCTCCTCTTCTGTACTCTAC/GATTGCGACCTCGATGTTGG | 950 |
| 16 | LR-J-12883/LR-N-13398 | CACCGGTTTGAACTCAGATC/CGCCTGTTTATCAAAAACAT | 550 |
| 17 | LR-J-12888/SR-N-14373 | ACGCTGTTATCCCTAAAGTA/AATCCACGATGTACCTTACT | 1500 |
| 18 | SR-J-14233/SR-N-14756 | AAGAGCGACGGGCGATGTGT/GACAAAATTCGTGCCAGCAGT | 550 |
| 19 | SR-J-14612/SR-N-14922 | AGGGTATCTAATCCTAGTTT/AAGTTTTATTTTGGCTTA | 300 |
| 20* | NEM-J-14741/ NEM-N-288 | TAAAAACTCACACAAAAATTTAC/GGTTAGGAGCTTGAATAGGTTTAG | 1400 |

* Species-specific primers designed in this study.
