# Supplementary material for: The First Mitochondrial Genome of the Sepsid Fly Nemopoda mamaevi Ozerov, 1997 (Diptera: Sciomyzoidea: Sepsidae), with Mitochondrial Genome Phylogeny of Cyclorrhapha
Source: PLoS One. 2015 Mar 31;10(3):e0123594. doi: 10.1371/journal.pone.0123594 (PMC4380458; doi:10.1371/journal.pone.0123594)
Supplement: S3 Table — (DOCX) [file pone.0123594.s003.docx]

**S3 Table.** AT%, GC%, AT-Skew and GC-Skew in complete Cyclorrhapha mt genomes.

| Species | A+T | G+C | AT-Skew | GC-Skew |
| --- | --- | --- | --- | --- |
| *Megaselia scalaris* | 76.8 | 23.2 | 0.013 | -0.181 |
| *Simosyrphus grandicornis* | 80.9 | 19.2 | -0.004 | -0.135 |
| *Fergusonina taylori* | 78.1 | 21.9 | 0.047 | -0.196 |
| *Liriomyza bryoniae* | 79.3 | 20.7 | 0.039 | -0.198 |
| *Liriomyza huidobrensis* | 78.3 | 21.7 | 0.037 | -0.171 |
| *Liriomyza sativae* | 77.5 | 22.5 | 0.048 | -0.173 |
| *Liriomyza trifolii* | 77.9 | 21.9 | 0.050 | -0.178 |
| *Bactrocera carambolae* | 73.6 | 26.5 | 0.065 | -0.223 |
| *Bactrocera correcta* | 73.2 | 26.8 | 0.063 | -0.224 |
| *Bactrocera cucurbitae* | 72.8 | 27.1 | 0.047 | -0.210 |
| *Bactrocera dorsalis* | 73.6 | 26.4 | 0.068 | -0.227 |
| *Bactrocera minax* | 67.2 | 32.7 | 0.131 | -0.315 |
| *Bactrocera oleae* | 72.6 | 27.4 | 0.088 | -0.277 |
| *Bactrocera papayae* | 73.5 | 26.4 | 0.067 | -0.227 |
| *Bactrocera philippinensis* | 73.5 | 26.3 | 0.067 | -0.224 |
| *Bactrocera tryoni* | 72.5 | 27.6 | 0.070 | -0.225 |
| *Ceratitis capitata* | 77.5 | 22.5 | 0.022 | -0.182 |
| *Procecidochares utilis* | 80.8 | 19.2 | 0.037 | -0.177 |
| *Drosophila littoralis* | 76.2 | 23.7 | 0.013 | -0.181 |
| *Drosophila melanogaster* | 82.2 | 17.9 | 0.017 | -0.151 |
| *Drosophila santomea* | 78.6 | 21.4 | 0.005 | -0.140 |
| *Drosophila yakuba* | 78.6 | 21.4 | 0.005 | -0.140 |
| *Nemopoda mamaevi* | 74.8 | 25.2 | 0.016 | -0.206 |
| *Haematobia irritans* | 79.1 | 21.0 | 0.004 | -0.124 |
| *Stomoxys calcitrans* | 78.9 | 21.1 | -0.001 | -0.137 |
| *Musca domestica* | 77.9 | 22.1 | 0.012 | -0.158 |
| *Scathophaga stercoraria* | 78.4 | 21.6 | 0.010 | -0.139 |
| *Calliphora vicina* | 77.6 | 22.4 | 0.021 | -0.179 |
| *Chrysomya albiceps* | 77.3 | 22.7 | 0.027 | -0.172 |
| *Chrysomya bezziana* | 75.9 | 24.1 | 0.033 | -0.212 |
| *Chrysomya megacephala* | 76.0 | 24.0 | 0.016 | -0.175 |
| *Chrysomya putoria* | 76.7 | 23.3 | 0.020 | -0.167 |
| *Chrysomya rufifacies* | 77.3 | 22.8 | 0.025 | -0.175 |
| *Chrysomya saffranea* | 76.4 | 23.5 | 0.018 | -0.174 |
| *Protophormia terraenovae* | 75.8 | 24.1 | 0.018 | -0.195 |
| *Cochliomyia hominivorax* | 76.9 | 23.1 | 0.035 | -0.203 |
| *Lucilia cuprina* | 77.6 | 22.3 | 0.018 | -0.166 |
| *Lucilia porphyrina* | 76.3 | 23.7 | 0.025 | -0.198 |
| *Lucilia sericata* | 77.6 | 22.3 | 0.015 | -0.166 |
| *Hemipyrellia ligurriens* | 77.3 | 22.6 | 0.022 | -0.177 |
| *Dermatobia hominis* | 77.8 | 22.2 | 0.044 | -0.225 |
| *Hypoderma lineatum* | 77.8 | 22.1 | 0.044 | -0.249 |
| *Sarcophaga impatiens* | 74.7 | 25.2 | 0.055 | -0.238 |
| *Sarcophaga peregrina* | 75.0 | 25.0 | 0.037 | -0.208 |
| *Elodia flavipalpis* | 80.0 | 20.0 | 0.003 | -0.150 |
| *Exorista sorbillans* | 78.4 | 21.5 | 0.020 | -0.172 |
| *Rutilia goerlingiana* | 77.7 | 22.3 | 0.040 | -0.229 |
