# Supplementary material for: The First Mitochondrial Genome of the Sepsid Fly Nemopoda mamaevi Ozerov, 1997 (Diptera: Sciomyzoidea: Sepsidae), with Mitochondrial Genome Phylogeny of Cyclorrhapha
Source: PLoS One. 2015 Mar 31;10(3):e0123594. doi: 10.1371/journal.pone.0123594 (PMC4380458; doi:10.1371/journal.pone.0123594)
Supplement: S4 Table — (DOCX) [file pone.0123594.s004.docx]

**S4 Table.** Codon usage of the *Nemopoda mamaevi* mt genome.

| Amino acid | Codon | N | RSCU | N+ | RSCU | N- | RSCU |
| --- | --- | --- | --- | --- | --- | --- | --- |
| Phe (F) | **UUU** | **294** | **1.72** | **158** | **1.55** | **136** | **1.99** |
|  | UUC | 47 | 0.28 | 46 | 0.45 | 1 | 0.01 |
| Leu^UUR^ (L) | **UUA** | **425** | **4.27** | **213** | **3.78** | **212** | **4.91** |
|  | UUG | 40 | 0.4 | 8 | 0.14 | 32 | 0.74 |
| Leu^CUN^ (L) | CUU | 60 | 0.6 | 53 | 0.94 | 7 | 0.16 |
|  | CUC | 5 | 0.05 | 5 | 0.09 | 0 | 0 |
|  | **CUA** | **60** | **0.6** | **52** | **0.92** | **8** | **0.19** |
|  | CUG | 7 | 0.07 | 7 | 0.12 | 0 | 0 |
| Ile (I) | **AUU** | **307** | **1.73** | **201** | **1.65** | **106** | **1.89** |
|  | AUC | 48 | 0.27 | 42 | 0.35 | 6 | 0.11 |
| Met (M) | **AUA** | **170** | **1.63** | **94** | **1.76** | **76** | **1.5** |
|  | AUG | 38 | 0.37 | 13 | 0.24 | 25 | 0.5 |
| Val (V) | GUU | 82 | 1.55 | 41 | 1.28 | 41 | 1.95 |
|  | GUC | 10 | 0.19 | 9 | 0.28 | 1 | 0.05 |
|  | **GUA** | **110** | **2.08** | **75** | **2.34** | **35** | **1.67** |
|  | GUG | 10 | 0.19 | 3 | 0.09 | 7 | 0.33 |
| Ser^UCN^ (S) | **UCU** | **114** | **2.73** | **70** | **3.06** | **44** | **2.33** |
|  | UCC | 6 | 0.14 | 5 | 0.22 | 1 | 0.05 |
|  | UCA | 94 | 2.25 | 63 | 2.75 | 31 | 1.64 |
|  | UCG | 11 | 0.26 | 6 | 0.26 | 5 | 0.26 |
| Pro (P) | **CCU** | **80** | **2.42** | **55** | **2.2** | **25** | **3.13** |
|  | CCC | 8 | 0.24 | 8 | 0.32 | 0 | 0 |
|  | CCA | 37 | 1.12 | 32 | 1.28 | 5 | 0.63 |
|  | CCG | 7 | 0.21 | 5 | 0.2 | 2 | 0.25 |
| Thr (T) | ACU | 84 | 1.78 | 57 | 1.61 | 27 | 2.3 |
|  | ACC | 16 | 0.34 | 14 | 0.39 | 2 | 0.17 |
|  | **ACA** | **84** | **1.78** | **68** | **1.92** | **16** | **1.36** |
|  | ACG | 5 | 0.11 | 3 | 0.08 | 2 | 0.17 |
| Ala (A) | **GCU** | **108** | **2.36** | **63** | **2.14** | **45** | **2.77** |
|  | GCC | 18 | 0.39 | 14 | 0.47 | 4 | 0.25 |
|  | GCA | 50 | 1.09 | 39 | 1.32 | 11 | 0.68 |
|  | GCG | 7 | 0.15 | 2 | 0.07 | 5 | 0.31 |
| Tyr (Y) | **UAU** | **131** | **1.56** | **56** | **1.29** | **75** | **1.85** |
|  | UAC | 37 | 0.44 | 31 | 0.71 | 6 | 0.15 |
| Stop (*) | **UAA** | **9** | **1.64** | **7** | **1.56** | **2** | **2** |
|  | UAG | 2 | 0.36 | 2 | 0.44 | 0 | 0 |
| His (H) | **CAU** | **48** | **1.19** | **35** | **1.06** | **13** | **1.73** |
|  | CAC | 33 | 0.81 | 31 | 0.94 | 2 | 0.27 |
| Gln (Q) | **CAA** | **61** | **1.79** | **46** | **1.92** | **15** | **1.5** |
|  | CAG | 7 | 0.21 | 2 | 0.08 | 5 | 0.5 |
| Asn (N) | **AAU** | **162** | **1.6** | **95** | **1.44** | **67** | **1.89** |
|  | AAC | 41 | 0.4 | 37 | 0.56 | 4 | 0.11 |
| Lys (K) | **AAA** | **56** | **1.35** | **32** | **1.52** | **24** | **1.17** |
|  | AAG | 27 | 0.65 | 10 | 0.48 | 17 | 0.83 |
| Asp (D) | **GAU** | **40** | **1.23** | **21** | **0.93** | **19** | **1.9** |
|  | GAC | 25 | 0.77 | 24 | 1.07 | 1 | 0.1 |
| Glu (E) | **GAA** | **69** | **1.73** | **45** | **1.91** | **24** | **1.45** |
|  | GAG | 11 | 0.28 | 2 | 0.09 | 9 | 0.55 |
| Cys (C) | **UGU** | **35** | **1.89** | **12** | **1.85** | **23** | **1.92** |
|  | UGC | 2 | 0.11 | 1 | 0.15 | 1 | 0.08 |
| Trp (W) | **UGA** | **93** | **1.86** | **66** | **1.89** | **27** | **1.8** |
|  | UGG | 7 | 0.14 | 4 | 0.11 | 3 | 0.2 |
| Arg (R) | CGU | 18 | 1.22 | 5 | 0.53 | 13 | 2.48 |
|  | CGC | 0 | 0 | 0 | 0 | 0 | 0 |
|  | **CGA** | **33** | **2.24** | **31** | **3.26** | **2** | **0.38** |
|  | CGG | 8 | 0.54 | 2 | 0.21 | 6 | 1.14 |
| Ser^AGN^ (S) | **AGU** | **55** | **1.32** | **20** | **0.87** | **35** | **1.85** |
|  | AGC | 3 | 0.07 | 3 | 0.13 | 0 | 0 |
|  | AGA | 50 | 1.2 | 16 | 0.7 | 34 | 1.8 |
|  | AGG | 1 | 0.02 | 0 | 0 | 1 | 0.05 |
| Gly (G) | GGU | 56 | 1 | 29 | 0.85 | 27 | 1.26 |
|  | GGC | 9 | 0.16 | 7 | 0.2 | 2 | 0.09 |
|  | **GGA** | **95** | **1.7** | **71** | **2.07** | **24** | **1.12** |
|  | GGG | 63 | 1.13 | 30 | 0.88 | 33 | 1.53 |
