# Supplementary material for: The First Mitochondrial Genome of the Sepsid Fly Nemopoda mamaevi Ozerov, 1997 (Diptera: Sciomyzoidea: Sepsidae), with Mitochondrial Genome Phylogeny of Cyclorrhapha
Source: PLoS One. 2015 Mar 31;10(3):e0123594. doi: 10.1371/journal.pone.0123594 (PMC4380458; doi:10.1371/journal.pone.0123594)
Supplement: S6 Table — (DOCX) [file pone.0123594.s006.docx]

**S6 Table.** Automated annotation results of the *Nemopoda mamaevi* mt genome by MITOS.

| **Name** | **Start** | **Stop** | **Strand** | **Length** |
| --- | --- | --- | --- | --- |
| trnI(gat) | 1 | 66 | + | 66 |
| trnQ(ttg) | 67 | 135 | - | 69 |
| trnM(cat) | 140 | 208 | + | 69 |
| nad2 | 230 | 1108 | + | 879 |
| trnW(tca) | 1237 | 1303 | + | 67 |
| trnC(gca) | 1296 | 1359 | - | 64 |
| trnY(gta) | 1360 | 1426 | - | 67 |
| cox1 | 1431 | 2939 | + | 1509 |
| trnL2(taa) | 2959 | 3024 | + | 66 |
| cox2 | 3030 | 3695 | + | 666 |
| trnK(ctt) | 3715 | 3785 | + | 71 |
| trnD(gtc) | 3789 | 3855 | + | 67 |
| atp8 | 3856 | 4014 | + | 159 |
| atp6 | 4005 | 4679 | + | 675 |
| cox3 | 4688 | 5470 | + | 783 |
| trnG(tcc) | 5483 | 5547 | + | 65 |
| nad3 | 5545 | 5892 | + | 348 |
| trnA(tgc) | 5900 | 5963 | + | 64 |
| trnR(tcg) | 5964 | 6026 | + | 63 |
| trnN(gtt) | 6029 | 6094 | + | 66 |
| trnS1(gct) | 6095 | 6162 | + | 68 |
| trnE(ttc) | 6163 | 6227 | + | 65 |
| trnF(gaa) | 6246 | 6311 | - | 66 |
| nad5_0 | 6334 | 8031 | - | 1698 |
| trnH(gtg) | 8047 | 8112 | - | 66 |
| nad4 | 8117 | 9451 | - | 1335 |
| nad4l | 9456 | 9743 | - | 288 |
| trnT(tgt) | 9746 | 9810 | + | 65 |
| trnP(tgg) | 9811 | 9876 | - | 66 |
| nad6 | 9879 | 10391 | + | 513 |
| cob | 10403 | 11527 | + | 1125 |
| trnS2(tga) | 11538 | 11604 | + | 67 |
| nad1 | 11643 | 12569 | - | 927 |
| trnL1(tag) | 12571 | 12635 | - | 65 |
| rrnL | 12596 | 13970 | - | 1375 |
| trnV(tac) | 13957 | 14028 | - | 72 |
| rrnS | 14027 | 14811 | - | 785 |
|  |  |  |  |  |
|  |  |  |  |  |
