# Supplementary material for: The First Mitochondrial Genome of the Sepsid Fly Nemopoda mamaevi Ozerov, 1997 (Diptera: Sciomyzoidea: Sepsidae), with Mitochondrial Genome Phylogeny of Cyclorrhapha
Source: PLoS One. 2015 Mar 31;10(3):e0123594. doi: 10.1371/journal.pone.0123594 (PMC4380458; doi:10.1371/journal.pone.0123594)
Supplement: S7 Table — (DOCX) [file pone.0123594.s007.docx]

**S7 Table.** Annotation results of the *Nemopoda mamaevi* tRNAs by tRNAscan-SE.

**Candidate tRNA Predictions in BED format:**

0 66 1.tRNA1-IleGAT 266 +

139 208 1.tRNA2-MetCAT 394 +

1236 1303 1.tRNA3-TrpTCA 271 +

2958 3024 1.tRNA4-LeuTAA 263 +

3714 3785 1.tRNA5-LysCTT 179 +

3788 3855 1.tRNA6-AspGTC 291 +

5482 5547 1.tRNA7-GlyTCC 321 +

5899 5964 1.tRNA8-AlaTGC 286 +

6028 6094 1.tRNA9-AsnGTT 239 +

6093 6163 1.tRNA10-PheAAA 127 +

6162 6227 1.tRNA11-GluTTC 289 +

9745 9810 1.tRNA12-ThrTGT 276 +

11537 11604 1.tRNA13-SerTGA 222 +

13375 13449 1.tRNA14-IleAAT 60 +

14459 14568 1.tRNA15-MetTAT 168 -

13956 14028 1.tRNA16-ValTAC 474 -

13335 13430 1.tRNA17-SupTTA 62 -

12570 12635 1.tRNA18-LeuTAG 309 -

9810 9876 1.tRNA19-ProTGG 258 -

8046 8112 1.tRNA20-HisGTG 248 -

6245 6311 1.tRNA21-PheGAA 209 -

3786 3855 1.tRNA22-ValGAC 190 -

1359 1426 1.tRNA23-TyrGTA 370 -

1295 1359 1.tRNA24-CysGCA 291 -

66 135 1.tRNA25-GlnTTG 369 -
